# Evaluating Oil Palm Trunk Biochar and Palm Oil as Environmentally Friendly Sustainable Additives in Green Natural Rubber Composites

**DOI:** 10.3390/polym17020223

**Published:** 2025-01-17

**Authors:** Narong Chueangchayaphan, Manop Tarasin, Wimonwan Phonjon, Wannarat Chueangchayaphan

**Affiliations:** Faculty of Science and Industrial Technology, Prince of Songkla University, Surat Thani Campus, Surat Thani 84000, Thailand; narong.c@psu.ac.th (N.C.); manop.ta@psu.ac.th (M.T.); phonjonwimonwan@gmail.com (W.P.)

**Keywords:** oil palm trunk, biochar, palm oil, sustainable additive, natural rubber, green composites

## Abstract

This research examines the possibility of palm oil and oil palm trunk biochar (OPTB) from pyrolysis effectively serving as alternative processing oils and fillers, substituting petroleum-based counterparts in natural rubber (NR) composites. Chemical, elemental, surface and morphological analyses were used to characterize both carbon black (CB) and OPTB, by using Fourier transform infrared spectroscopy (FTIR), X-ray diffraction (XRD), Brunauer–Emmett–Teller (BET) gas porosimetry, and scanning electron microscopy (SEM). The influences of OPTB contents from 0 to 100 parts per hundred rubber (phr) on thermal, dielectric, dynamic mechanical, and cure characteristics, and the key mechanical properties of particulate NR-composites were investigated. OPTB enhanced the characteristics of the composites, as demonstrated by a rise in dielectric constant, thermal stability, storage modulus, glass transition temperature (T_g_), hardness and modulus at 300% elongation, along with a decrease in the loss tangent (tan δ). Tear strength exhibited an increase with OPTB content up to a specific threshold, whereas tensile strength and elongation at break declined. This implies a compromise between the various mechanical properties when incorporating OPTB as a filler. This work supports the potential application of OPTB as a renewable substitute for CB in the rubber industry, particularly in tire production and other industrial rubber applications, which would also bring environmental, sustainability, and economic benefits for the palm oil-related industry.

## 1. Introduction

Biopolymers have gained a lot of attention in recent years due to their potential to lessen the need for fossil fuel resources and nonbiodegradable polymers, both of which contribute significantly to environmental pollution [1]. Natural rubber (NR), derived from the *Hevea brasiliensis* plant, is a naturally occurring polymer consisting of a combination of poly(*cis*-1,4-isoprene) and biological components. This unique composition imparts distinct characteristics to the material [2]. Thus, there has been a consistent rise in the significance of NR for human beings due to its inherent qualities of elasticity, low hysteresis, high strength, exceptional toughness, and versatile production capabilities [3]. However, it should be noted that raw NR possesses a soft texture and exhibits relatively low mechanical properties. Consequently, the incorporation of additives and the process of vulcanization have become imperative in order to enhance its overall performance [4]. Processing oil is an additive needed in NR compounds because it acts as an internal lubricant to achieve acceptable processability, improves flex life at a low temperature, filler dispersion, end-use properties, and gives a cost benefit [5,6]. Vegetable oil has replaced petroleum-based oil for decades because of its broad availability, non-toxicity, affordability, environmental sustainability, and being a renewable resource [7]. Vegetable oils have been extensively studied as alternative processing aids in rubber compounds due to their environmental benefits and ability to replace petroleum-based oils. Castor oil has been investigated as a plasticizer in natural rubber (NR), showing improved processability and mechanical properties [8]. Similarly, the natural oils soybean, palm, and sunflower oil have been explored for their effectiveness as processing aids and activators in carbon black-filled NR [9]. Soybean oil and its modified forms have demonstrated potential in replacing petroleum oils across various elastomers, including NR, styrene-butadiene rubber (SBR), and ethylene-propylene-diene monomer rubber (EPDM), contributing to enhanced filler dispersion, processing characteristics, and mechanical performance [10,11,12,13,14,15]. Palm oil, a readily available and renewable resource, has also been shown to improve the processing and mechanical properties of EPDM and silica-filled NR composites [16,17]. In nitrile butadiene rubber (NBR), palm-based processing aids have optimized curing systems and physical properties [18]. These findings highlight the versatility and potential of vegetable oils to advance sustainable practices in rubber manufacturing. In this study, palm oil was selected as the processing oil for the fabrication of green composite natural rubber. This selection was based on a review of the relevant literature, highlighting its status as the most extensively manufactured vegetable oil globally, accounting for a substantial share of the vegetable oil industry and offering a reasonable price for large-scale industrial applications [19,20]. Palm oil is derived from renewable resources and contains key fatty acids, including palmitic, oleic, and linoleic acids [21], which contribute to its excellent lubricating properties, high heat resistance [9] and compatibility with rubber matrices. Additionally, its renewable and environmentally friendly nature aligns with the objectives of developing sustainable and eco-friendly rubber composites. These factors collectively support the suitability of palm oil as an effective alternative to petroleum-based processing oils in rubber compounding.

Filler is one of the compounding ingredients required to enhance the properties of rubber, and common ones are carbon black, clay, calcium carbonate, silica, etc. [22]. The type of filler most frequently utilized in the rubber industry as a reinforcing substance is carbon black (CB) [23]. Despite the advantageous physical, mechanical, thermal, and barrier qualities that CB imparts to rubber composites, it is not without its drawbacks. CB is produced from the incomplete combustion of heavy petroleum products. The incomplete combustion of petroleum products can result in the emission of poisonous fumes and the generation of hazardous waste [24]. As a result, the production and utilization of CB give rise to numerous risks of health complications and environmental damage. Thus, it is imperative that the rubber industry adopts alternative, renewable, and environmentally favorable filler materials in place of CB. Biochar is a solid carbon-rich substance like CB; it is obtained by subjecting biomass to elevated temperatures in a sealed chamber with varying degrees of oxygen lack. In this controlled environment, biomass undergoes a thermochemical transformation, producing biochar. Because of its affordability and environmentally friendly nature, biochar has garnered significant interest for diverse uses such as enhancing soil quality, managing waste, mitigating greenhouse gas emissions, and generating energy [25,26]. Biomass derived from oil palm plantations presents itself as a highly promising and viable option as fuel and feedstock of carbon black, serving as a commendable alternative to conventional renewable and sustainable resources.

Palm oil is the predominant oil produced on a global scale, playing a substantial role in the vegetable oil market [20]. The increasing worldwide need for palm oil has resulted in intensified production in the oil palm estates. This heightened cultivation has resulted in the significant production of substantial amounts of biomass from milling operations and plantations. Among the various categories of residual oil palm biomass are empty fruit bunches (EFBs), palm kernel shells (PKSs), mesocarp fiber (MF), oil palm fronds (OPFs), and oil palm trunks (OPTs). OPTs hold significant appeal due to their consistent year-round availability following the felling of oil palm trees [27,28]. The substantial volume of OPTs generated poses inevitable challenges in waste management. Instead of resorting to landfill disposal or open burning, which result in significant air pollution, there is an opportunity to transform this waste into value-added materials. To achieve a significant reduction in the organic compound content, OPT is subjected to pyrolysis to generate biochar. In contrast to unprocessed biomass, biochar is more advantageous owing to its superior qualities, which include reduced moisture content, elevated calorific value, decreased density for storage purposes, and long-lasting durability [29]. This approach can effectively address and mitigate waste disposal concerns. Other than its application, as previously mentioned, the biochar produced possesses the ability to serve as an additive in rubber composites. This would provide an alternative to carbon black, which is derived from petroleum. In the prior literature, there are reports on the utilization of biochar from oil palm biomass as bio-filler in rubber composites, for example the incorporation of palm kernel shell biochar (PKSB) in NR composites [22,30,31,32] and in carboxylated nitrile butadiene rubber (XNBR) [22]. The viability of using OPTB as a flame-retardant component has been examined in two varieties of specialty natural rubber (SpNR) latex foam, epoxidized and deproteinized, providing a limited study of the application of OPTB as a filler in vulcanized natural rubber [33].

The concept of a sustainable circular economy has gained significant attention for its economic, social, and environmental benefits. By utilizing green and renewable materials, it supports industrial applications that benefit both current and future generations [19]. This study is innovative in simultaneously utilizing two renewable materials—OPTB as a filler and palm oil as a processing oil—to replace carbon black- and petroleum-based processing oils in NR composites. To our knowledge, no previous research has combined these materials in a single formulation. The functional groups, chemical composition, and morphology of OPTB were characterized using FTIR, XRD, and SEM. The effects of OPTB content (0–100 phr) on the cure characteristics and thermal, dynamic mechanical, dielectric, and mechanical properties of NR composites were analyzed. Comparative samples with CB as a filler were prepared using the same methodology. This approach offers a significant advance in reducing fossil fuel consumption and developing eco-friendly additives by transforming agricultural waste into high-value materials, aligning with sustainability and circular economy principles.

## 2. Materials and Methods

### 2.1. Materials

Palm oil was obtained from Suksomboon Vegetable Oil Co., Ltd. (Chonburi, Thailand). Standard Thai Rubber (STR20) was used in the rubber formulation and the other chemicals were N-phenyl-p-phenylenediamine (6PPD), zinc oxide (ZnO), stearic acid (SA), diphenyl guanidine (DPG), 2-Mercaptobenzothiazyl disulfide (MBTS), carbon black (N550), and sulfur (S), supplied by Bossoftical Public Co., Ltd. (Songkhla, Thailand). All the chemicals were utilized as received, without further purification. OPTs were gathered from a nearby palm plantation located in Surat Thani, Thailand. OPTs used in this study were from the Surat Thani 2 cultivar, harvested from a 22-year-old plantation located in Mueang District, Surat Thani, Thailand. The palms had an average trunk height of 9.5 m. For the experiment, the trunks were cut into 10 cross-sectional discs (thickness: 5 cm each) at intervals of 1 m along the length of the trunk to ensure a representative sampling of the material. These discs were pre-dried at 100 ± 5 °C in a controlled oven until their weights stabilized, which is essential for achieving efficient pyrolysis and minimizing energy consumption during the process. The pre-dried samples were then arranged evenly in the pyrolysis chamber to optimize heat distribution and ensure uniform carbonization. The OPTs were heated gradually from ambient temperature and held at 600 °C for 6 h. The resulting pyrolytic OPTB was allowed to cool for 24 h before being ground using a grinder. Following this, the ground material was sifted through a 250-mesh sieve and dried in an oven at 105 °C until no further changes in quality were observed.

### 2.2. Characterization of CB and OPTB Powder

FTIR spectra were obtained using a Vertex70 FTIR spectrometer (Thermo Scientific, Bruker, Ettlingen, Germany) with the KBr pellet technique. XRD analysis was conducted with Cu-Kα radiation, operating at 40 kV and 30 mA, scanning from 10° to 90° (2θ), and equipped with a 0.154 monochromator. Specific surface area was determined using Brunauer–Emmett–Teller (BET) gas porosimetry (ASAP2060, Micromeritics, Norcross, GA, USA) employing the BET N2 method. Particle size and morphology of CB and OPTB powders were analyzed using a particle size analyzer (Beckman Coulter LS 230, Vernon Hills, IL, USA) and SEM (QUANTA400, Thermo Fisher Scientific, Brno, Czech Republic), respectively. Prior to SEM imaging, a thin layer of gold was sputter-coated onto the surfaces of CB and OPTB powders under a vacuum.

### 2.3. Preparation and Characterizations of NR Composites

The compositions of the compounds, expressed in parts per hundred of rubber (phr), are detailed in Table 1.

NR composites were prepared by blending in a two-roll mill for 30 min. Following sheeting out, the compound was left to rest for 16–24 h before undergoing vulcanization at 160 °C utilizing an electrically heated plate press (Charoen Tut Co., LTD., Bangkok, Thailand) operating at a pressure of 1500 psi for the optimal curing time determined using a moving die rheometer (MDR) (Alpha-Technologies Co., Ltd., Hudson, OH, USA) in accordance with ASTM D5289 standards, resulting in 2 mm thick sheets.

### 2.4. Characterization of NR Composites

#### 2.4.1. Rheological Property Measurement

The Mooney viscosity (ML(1 + 4), 100 °C) was determined at 100 °C using a large rotor for a duration of 4 min following 1 min of preheating. Results were recorded as ML(1 + 4) and obtained using a MonTech Mooney viscometer (MV2020) in compliance with ASTM D1646 standards. The Payne effect assessment was conducted utilizing a rubber process analyzer (RPA2000, Alpha Technologies, Hudson, OH, USA) operating in the strain sweep mode. This involved evaluating the storage modulus of uncured NR composites at various strain amplitudes. Strain sweep tests were employed to measure the storage shear moduli (G_0_) of samples across different shear strain amplitudes, ranging from 0.56 to 1000%, with a fixed oscillation frequency of 1 Hz at a temperature of 100 °C.

#### 2.4.2. Thermal and Dynamic Mechanical Properties

Thermogravimetric analysis (TGA/DSC 3+ model, Mettler Toledo, Greifensee, Switzerland) was conducted using a simultaneous thermal analyzer, employing a heating rate of 10 °C/min within the temperature range from 25 to 1000 °C under a nitrogen atmosphere. Dynamic mechanical thermal analysis was performed using a PerkinElmer DMTA 8000. Samples were subjected to tension mode testing from −100 to 150 °C at a heating rate of 3 °C/min, with fixed frequency and strain set at 10 Hz and 0.1%, respectively.

#### 2.4.3. Dielectric and Mechanical Properties

The dielectric constant (ε′) was determined using an impedance analyzer across the frequency range of 10^3^–10^6^ Hz at an AC potential of 1 V. Durometer (Instron, Norwood, MA, USA) measurements were employed to assess the hardness of 6 mm thick samples in accordance with ASTM D2240 standards. Tear and tensile tests were conducted using a universal tensile testing machine (Tinius Olsen, model 10ST, Salfords, UK) on dumbbell-shaped test specimens prepared according to ASTM D624 and ASTM D412, respectively, at a crosshead speed of 500 mm/min. For each test, five duplicate samples were used to ensure the accuracy and reproducibility of the results.

## 3. Results and Discussion

### 3.1. Characterization of CB and OPTB

**Figure 1:**
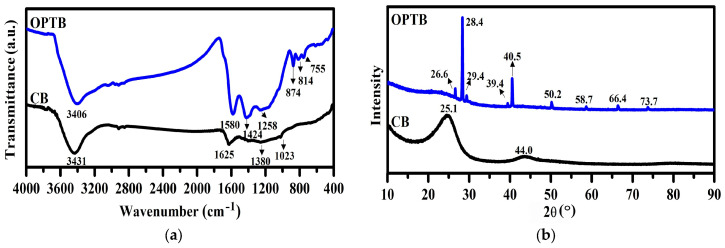
(**a**) FTIR spectra and (**b**) XRD analyses of CB and OPTB.

Pyrolytic chars are the most economically viable substitutes for carbon black in the rubber industry, used as carbon fillers. To assess feasibility, a pyrolytic OPTB was analyzed and compared to a commercial CB, N550. The FTIR analysis was performed in order to ascertain the chemical functional groups present in both CB and OPTB, as shown in Figure 1a. It is evident that the weaker peaks in both cases, located between 2800 and 2975 cm^−1^, which are attributed to the stretching vibrations of -CH bonds [34], could be the consequence of pyrolysis-induced alkane breakdown resulting in the significantly reduced hydrogen content of the carbons [35]. In addition, the prominent absorption peak at approximately 3400 cm^−1^, corresponding to the stretching vibrations of -OH of hydroxyl, indicates carboxylic and phenolic functional groups present in both the materials [36]. For the case of CB, the peak at around 1625 cm^−1^ was exclusively seen, and it is attributed to C=C stretching vibrations [37,38]. Meanwhile, Zang et al. (2023) ascribed the observed peaks to the stretching of C=O bonds, indicating the presence of carboxyl, ketenes, lactones [39], and pyrones [40]. In addition, the CH stretching of the aromatic molecule was shown by the weak intensity absorption at 1380 cm^−1^ [38]. The vinyl ether was detected at a wavenumber of 1023 cm^−1^, corresponding to the stretching vibrations of the C-O bond. The spectrum for OPTB exhibited clearly visible bands at 1580 cm^−1^, attributed to an aromatic skeleton vibration of the C–C bond [41]. This signifies the aromatic ring of lignin [42,43]. According to Zappielo et al. (2016), a noticeable peak around 1580 cm^−1^ indicates the presence of carbonyl or hydroxyl groups. The absorption bands at 1424 cm^−1^ represent C–H deformation by the bending of alkanes or alkyl groups, while 1258 cm^−1^ is for the C–O–C stretching of ester groups [37]. The peaks detected at 874, 814, and 755 cm^−1^ likely originated from the bending of C–H bonds in aromatic rings [44,45]. The aforementioned results show that, while the FTIR peaks of the two raw materials, OPTB and CB, were slightly different, the chemical structures of CB and OPTB had certain identical functional groups, such as C=C stretching in aromatic and oxygen-containing groups, including carboxylic, hydroxyl, phenolic (hydroxyl), carbonyl, lactone, carboxylic acid anhydride, and cyclic peroxide groups, as reported by [46,47,48]. This was most likely caused by the varied sources and methods used to prepare OPTB and CB.

XRD was used to characterize the CB and OPTB fillers, as shown in Figure 1b. It was found that CB demonstrated typical amorphous carbon in the diffractograms. The carbons show no signs of prominent Bragg peaks caused by mineral impurities. Nevertheless, a broad diffraction peak at 2θ values ranging from 20 to 30° (002) and 40 to 50° (100) indicates the existence of graphitic carbon alongside amorphous carbon [34,35,36,49,50]. In contrast, the XRD patterns of OPTB exhibited several sharp diffraction peaks reflecting different mineralogical phases, indicating miscellaneous mineral crystals and other inorganic components. It was noted that several peaks overlapped and provided multiple-peak combinations. The major crystalline peaks of the OPTB were for potassium chloride (KCl), silicon oxide (SiO_2_), and calcium carbonate (CaCO_3_). These mineral elements (silicate, potassium salt, and calcium carbonate) made up the majority of the ash that was released as the cellulose and lignin components of OPT were progressively broken down [51].

The BET surface area of the CB that was determined in this investigation (37 m^2^/g) was comparable to that which previous studies have found [40]. The BET specific surface area of OPTB was higher than that of CB, as shown in Table 2. Despite this, the CB particles demonstrated smaller diameters, not only in the mean value (D_50_), but also for 10% and 90% thresholds in sample volume (D_10_ and D_90_, respectively) when compared to OPTB. Apparently, the abundant inorganic materials and metal elements in OPTB (shown in XRD analysis) generated pores or catalytically accelerated the creation of pores in the carbonaceous material [36].

**Figure 2:**
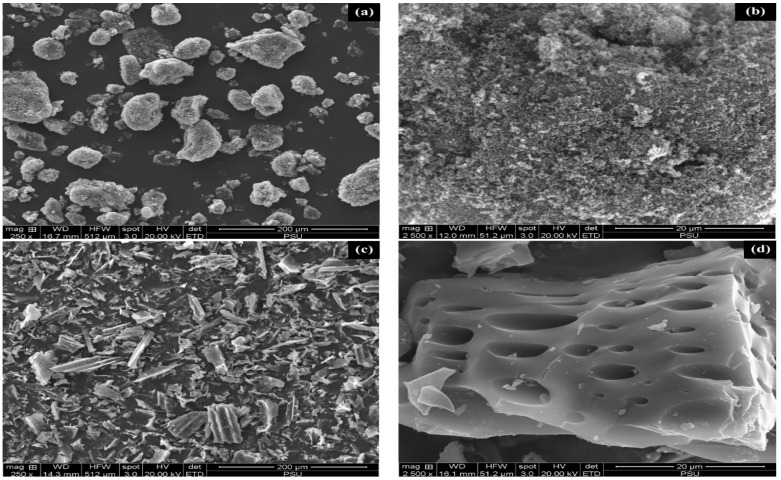
SEM images of CB and OPTB showing the morphologies of (**a**) CB at ×250 magnification, (**b**) CB at ×2500 magnification, (**c**) OPTB at ×250 magnification, and (**d**) OPTB at ×2500 magnification.

Figure 2 provides SEM images of CB and OPTB, demonstrating significant differences between the samples. It was found that CB showed different shapes of carbon black aggregates, often spheroidal or ellipsoidal with a rough surface. OPTB had larger sized particles with pores on their surfaces. The images distinctly depict the porous structure of OPTB, with pores evident on the surface and displaying discernible sizes and shapes of both cylindrical and polygonal cross-sections. The formation of these porous channels resulted from the gradual breakdown of the lignocellulosic constituents, cellulose, hemicelluloses, and lignin. The residual non-volatile components were transformed into biochar [52]. It is the porosity of the larger particles in OPTB that explains its larger specific surface.

### 3.2. Thermal Stability Study

**Figure 3:**
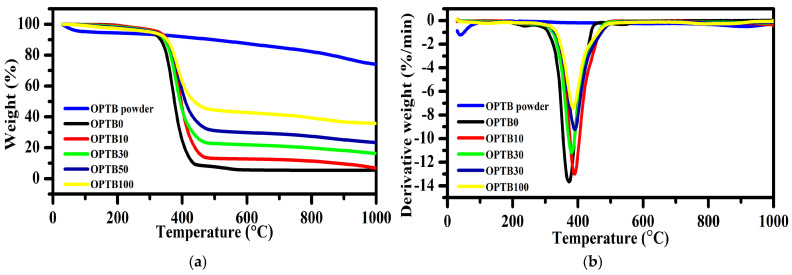
Thermal properties of OPTB powder and NR composites: (**a**) TGA curves and (**b**) DTG curves.

The influence of OPTB content on NR composites is shown in Figure 3. The thermal behavior of all specimens showed mutual resemblance, characterized by a primary decomposition peak. It can be noted that the TGA curves gradually declined until reaching a stage where the NR composites started to decompose. Thermal degradation of pure NR occurred between 195 and 430 °C, with T_max_ around 373 °C. It was also observed that the T_max_ slightly shifted to higher temperatures with increasing OPTB content, as listed in Table 3. This suggests that adding OPTB to NR composites increased their thermal stability. Pure NR produced the least final residue at 600 °C among the composites examined in the study. The ultimate residual amount rose with increasing OPTB content, attributable to the buildup of a greater amount of char capable of enduring high temperatures. Moreover, OPTB exhibits a slower decomposition rate in comparison to NR. Consequently, with the rise in OPTB concentration, a greater residual weight was noted [33].

### 3.3. Dielectric Properties

**Figure 4:**
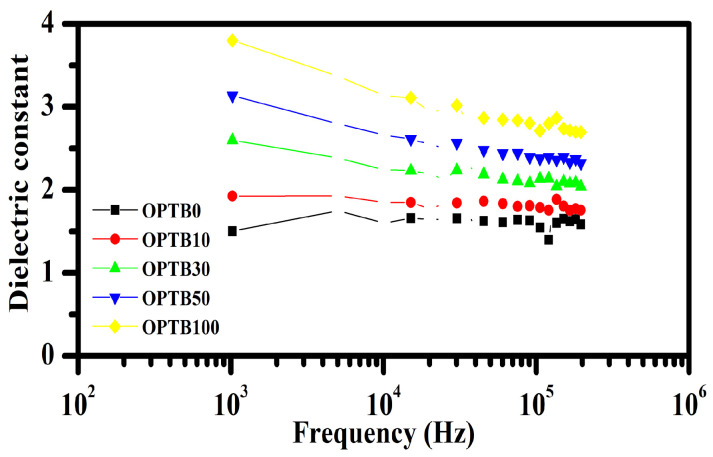
Dielectric constants of NR composites by frequency of excitation.

Figure 4 depicts the relative dielectric constant for NR composites with variable OPTB contents, at various frequencies and environmental conditions. It can be noted that as frequency increased, the dielectric constant declined across all NR composites. This is because the polarization of the specimen is unable to synchronize with a high-frequency electric field [53], as well as a gradual reduction in dipole movement or alteration in orientation. Pure NR exhibited the lowest dielectric constant (1.50 at 1 kHz) due to its hydrophobic (nonpolar) structure. The addition of OPTB raised the dielectric constant of NR composites over the whole frequency range. For example, 100 phr OPTB-NR composite demonstrated the highest dielectric constant (3.80 at 1 kHz), for an increase by 153%, suggesting that OPTB is more prone to polarization by an electric field than NR. The increased dielectric constant can be due to the significant interfacial polarization in OPTB/NR composites. Interfacial polarization is generated by charge accumulation at the phase interfaces of the filler (here biochar) and rubber matrix [54]. In addition, the presence of carbonaceous elements within biochar could be pivotal in fostering significant polarization within the material. The FTIR results demonstrated π electrons found in carbon materials, which have the freedom to move across wide areas, presenting a particularly intriguing phenomenon [55]. Moreover, the dielectric constant increased with OPTB loading due to the mineral phases identified by XRD. As OPTB content rises, the mineral concentration in the matrix increases, resulting in more charge carriers for polarization and a higher dielectric constant.

### 3.4. Dynamic Behaviors

**Figure 5:**
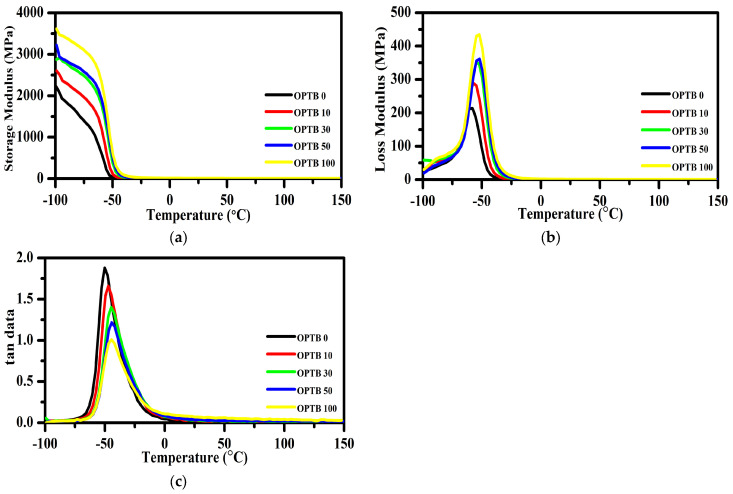
(**a**) Storage modulus, (**b**) loss modulus, and (**c**) tan δ for the experimental NR composites.

It is commonly known that elastic modulus and stored energy are both quantified by the storage modulus. The temperature dependence of storage modulus is reported in Figure 5a. The results show that when temperature increased, the storage modulus generally decreased, because chain mobility increased and physical bonding and chain entanglements decreased [56]. The NR composite with 100 phr OPTB had the highest storage modulus in the glassy region. When OPTB was introduced to NR composites, the storage modulus rose in comparison to pure NR. This is because the NR chain motion is restricted by the high-stiffness OPTB filler.

The loss modulus (E″) represents the viscous response and damping as a result of energy dissipation as heat. The addition of OPTB to the NR matrix broadened the loss modulus peak, as seen in Figure 5b. This could be explained by the fact that the addition of filler results in a larger number of chain segments inside the composites, which inhibits relaxation [57].

Figure 5c depicts the temperature-dependent evolution of the damping factor, also known as tan δ (loss to storage modulus ratio). The OPTB in NR composites resulted in a much lower height of the tan δ peak, indicating a lower lost-to-stored energy ratio in the T_g_ zone. The decrease in tan δ_max_ may be attributed to the dense and entangled OPTB filler network, which significantly hinders chain mobility during glass transition. It has been documented that natural rubber is among the polymer materials exhibiting the highest damping factor, attributed to the inherent movement of the polyisoprene backbone. The presence of additional materials in its matrix tends to limit damping capacity [56].

The transition to the maximum of a tan δ peak is closely related to the glass transition temperature T_g_. The tan δ peak parameters are summarized in Table 3. The glass transition temperatures of NR composites were impacted by the addition of OPTB. When OPTB was added to NR composites, a shift in T_g_ to a higher temperature was noted. It resulted from filler–rubber interactions limiting the mobility of the rubber chains. The rolling resistance and wet traction properties of NR are extremely significant for car tire applications. It is common practice to use the dynamic mechanical loss angle tan δ at 60 °C of vulcanized compounds as a predictor of rolling resistance. As a result, a low tan δ at 60 °C is of importance, since it indicates that the vulcanized compound is performing better. When rolling resistance is reduced, less energy is needed to maintain the speed of the vehicle, which conserves fuel for the internal combustion engine and lowers the emissions. Results show that the rolling resistance of NR composites tended to decrease with OPTB content. The energy loss of the rubber vulcanizate, or tan δ at 0 °C, is used to assess the good wet-grip qualities. A high tan δ at 0 °C indicates good wet-grip. The highest tan δ at 0 °C was recorded for 100 phr OPTB filled NR composite, while tan δ at 0 °C was lower or the wet-grip characteristics were poorer for pure NR.

**Figure 6:**
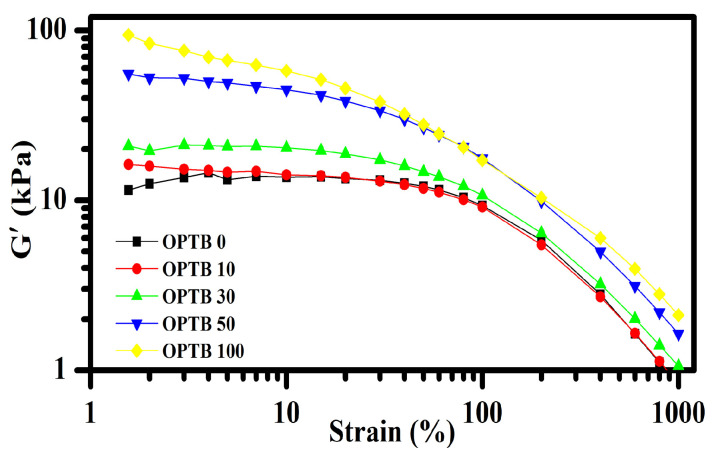
Storage modulus vs. strain for the NR composites.

It is well known that the addition of fillers to a rubbery matrix significantly alters its viscoelastic behavior. The Payne effect characterizes alterations in the characteristics of an elastomeric component in relation to the applied strain. It can be explained in terms of a filler–filler network in the rubber matrix. The variation in storage modulus of NR filled with varying loadings of OPTB as a function of dynamic strain amplitude and a summary of findings are demonstrated in Figure 6 and Table 4. It was found that the storage modulus decreased with strain, which is attributed to the filler–filler network breaking down at larger dynamic strains. In addition, the Payne effect increased with OPTB content. The maximum storage modulus and Payne effect magnitude were observed for NR composite filled at 100 phr with OPTB, indicating the strongest effects of a filler–filler network in the elastomer matrix. This is, however, an excessive filler content causing a loss of key material properties.

### 3.5. Rheological Properties

**Figure 7:**
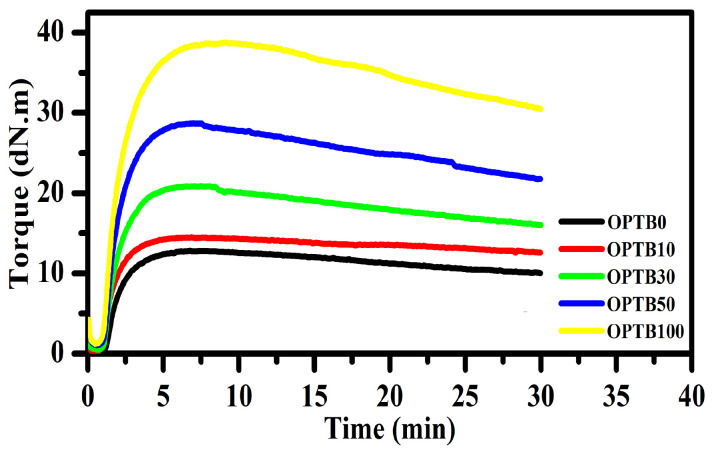
Time profiles of torque for the NR compounds.

The rheological responses of NR compounds, acquired at 160 °C using an MDR rheometer, are displayed in Figure 7, and the summarizing data for scorch time (t_s1_), cure time (t_c90_), cure rate index (CRI), minimum torque (M_L_), maximum torque (M_H_), and delta torque (M_H_–M_L_) are given in Table 5. t_s1_ for pure NR and NR composites were similar, with the exception of the 100 phr OPTB case, which had a 50% decrease in t_s1_ compared to pure NR. On the other hand, t_c90_ displayed a marginally rising trend with OPTB content. This may be because the surface activity of OPTB, which includes oxygen-containing compounds such as hydroxyl functional groups (see the previously discussed FTIR analysis of OPTB), retards t_c90_. The M_L_ and M_H_ denote viscosity and rigidity, as well as the stiffness of the rubber compound, respectively. It was found that the characteristics for the vulcanization of all prepared rubber compounds had the same trend of increasing M_L_ and M_H_ with filler content, because the filler was limiting the molecular motions of NR chains. These results are supported by the Mooney viscosity of unvulcanized rubber, often influenced by the macrostructure and flow properties of the rubber composite. It revealed that a rise in Mooney viscosity in unvulcanized rubber composites was associated with an increase in OPTB content, attributed to the hydrodynamic impact of the filler within the rubber. The crosslink density from both chemical and physical crosslinks can be assessed from delta torque (M_H_–M_L_). Pure NR exhibited the least M_H_–M_L_, whereas OPTB-filled NR composites displayed a rising trend with OPTB loading. This could be attributed to crosslinking between filler and NR matrix phases.

### 3.6. Mechanical Properties

**Figure 8:**
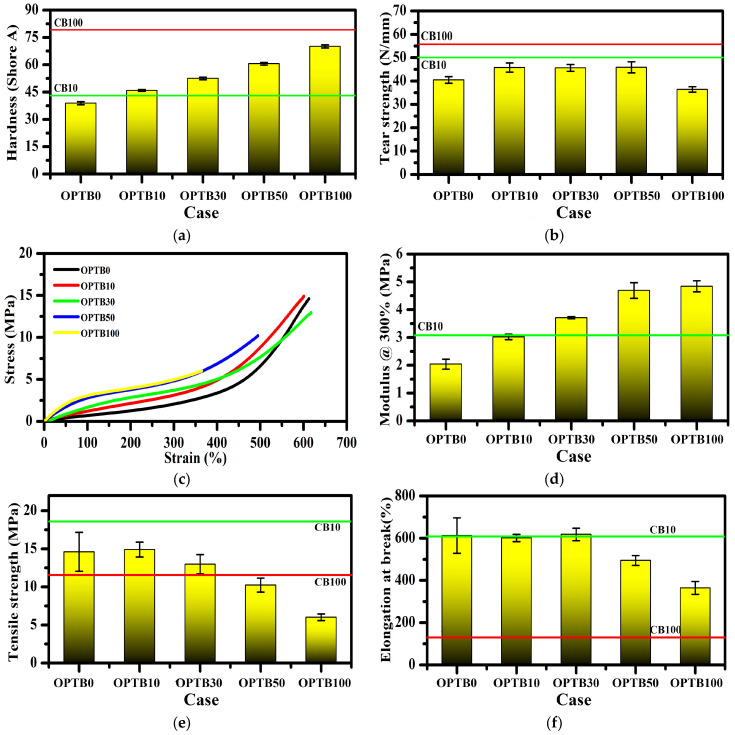
Physical and mechanical properties of NR composites: (**a**) hardness, (**b**) tear strength, (**c**) stress–strain curves, (**d**) modulus @300%, (**e**) tensile strength, and (**f**) elongation at break.

The incorporation of OPTB resulted in increases in hardness (Figure 8a), modulus at 100% elongation (M100), and modulus at 300% elongation (M300) (Figure 8d) in the OPTB filled NR composites. This can be ascribed to the notably high hardness and modulus of OPTB, along with the strong interfacial adhesion between OPTB filler and NR matrix. Consequently, OPTB particles could proficiently resist the load, enhancing the indentation resistance of the composites [58]. The tear strength exhibited an increasing trend with OPTB loading up to 70 phr (Figure 8b). This enhancement can be attributed to improved interfacial adhesion between the OPTB filler and NR matrix. The adhesion mechanism involves hydrogen bonding between the carboxylic (-COOH) and phenolic (-OH) groups present on the OPTB surface and the proteins and phospholipids found in the non-rubber components of NR, as evidenced by the FTIR analysis discussed previously. However, the tear strength shows a significant decrease when OPTB loading exceeds 70 phr, reaching its lowest value at 100 phr. Obviously, the tensile strength decreased gradually with OPTB loading, from pure NR onwards (Figure 8e). Nevertheless, incorporating CB enhanced tensile strength, attributed to the interactions between CB filler and rubber matrix, alongside its heightened activity and smaller particle size compared to OPBT [4]. The sizable particles of OPTB induced localized stresses within the composites, which undermined the strength of the composite, in particular reducing the tensile strength [59]. Furthermore, the greater particle size of OPTB may prevent the strain-induced crystallization of NR, also reducing tensile strength. This is the opposite effect to that of adding biochar to synthetic rubber [4,22,60,61]. There are studies seeking to improve the tensile strength of biochar/polymer (or rubber) composites by reducing the particle size of biochar [62,63]. When OPTB was increased to 100 phr, there was a significant reduction in tensile strength and tear strength. This indicates an excessive filler content that caused the filler to aggregate, hindering appropriate filler–matrix interactions and forming stress-concentration points initiating fracture failures. A substantial incorporation of OPTB markedly reduced the elongation at break of the NR composites (Figure 8f), signifying diminished ductility and toughness. Typically, as filler content increases, ductility and toughness decrease due to the filler particles serving as hindrances, impeding the slippage and movement of rubber chains, and consequently reducing ductility [58].

## 4. Conclusions

This study investigated the feasibility of using pyrolytic OPTB as a replacement for commercial CB in the rubber industry. FTIR analysis revealed the presence of similar functional groups in both CB and OPTB, including C=C and oxygen-containing groups. However, XRD analysis showed that CB possessed an amorphous structure, while OPTB contained various crystalline mineral phases. The addition of OPTB significantly enhanced key composite characteristics. The glass transition temperature (T_g_) increased by approximately 5.7 °C, indicating reduced polymer chain mobility and greater rigidity due to the filler’s reinforcing effect. Thermal stability improved by 13 °C, reflecting enhanced heat resistance, while the dielectric constant rose by about 153%, demonstrating improved electrical energy storage capabilities. However, the mechanical properties were not uniformly improved. Hardness, modulus, and tear strength increased with OPTB contents up to a certain point, while tensile strength and elongation at break decreased. This suggests a trade-off between the various mechanical properties when using OPTB as a filler. Overall, the results suggest that OPTB has the potential to partly replace CB in rubber composites. Nonetheless, challenges remain, including optimizing filler content and particle size to achieve a better balance of properties. Additionally, the large-scale adoption of OPTB faces hurdles such as feedstock variability, high production costs, and resistance from industries reliant on carbon black. Addressing these issues through precise process control and sustainable practices is essential for establishing OPTB as a competitive, eco-friendly filler.

## Figures and Tables

**Table 1:** Formulations used for NR composites with, alternatively, OPTB or CB filler.

Component	Case of OPTB (phr)				
	OPTB0	OPTB10	OPTB30	OPTB50	OPTB100
STR20	100	100	100	100	100
ZnO	5	5	5	5	5
SA	1	1	1	1	1
6PPD	1	1	1	1	1
Palm oil	10	10	10	10	10
MBTS	1	1	1	1	1
DPG	0.5	0.5	0.5	0.5	0.5
C	2.5	2.5	2.5	2.5	2.5
OPTB or CB	0	10	30	50	100

**Table 2:** Specific surface areas (from BET) and particle size distribution statistics for CB and OPTB.

Parameter	CB	OPTB
S_BET_ (m^2^/g)	37.06	68.09
D_10_ (µm)	0.5	6.4
D_50_ (µm)	2.2	17.9
D_90_ (µm)	7.2	35.7

**Table 3:** Thermal properties and dynamic mechanical properties of NR composites according to DMA and TGA.

Case	DMA			TGA	
	tan δ (at 0 °C)	tan δ (at 60 °C)	T_g_ (°C)	T_max_ (°C)	Residue (%)
OPTB0	0.047	0.000	−50.20	373	5.46
OPTB10	0.076	0.004	−47.15	390	6.85
OPTB30	0.074	0.013	−44.75	381	16.31
OPTB50	0.082	0.023	−44.30	391	23.41
OPTB100	0.108	0.049	−44.50	386	35.70

**Table 4:** Payne effect for the NR composites.

Case	G′_1.56_	G′_100_	G′_1000_	∆G′ = G′_1.56_−G′_100_	∆G′ = G′_1.56_−G′_1000_
OPTB0	11.49	9.31	0.79	2.18	10.7
OPTB10	16.27	9.13	0.83	7.14	15.44
OPTB30	20.94	10.71	1.06	10.23	19.88
OPTB50	55.51	17.65	1.64	37.86	53.87
OPTB100	94.32	17.25	2.11	77.07	92.21

**Table 5:** Cure characteristics and Mooney viscosities of the NR compounds.

Case	Mooney Viscosity (MU)	M_L_ (Ib/In)	M_H_ (Ib/In)	M_H_–M_L_ (Ib-In)	t_s1_ (min)	t_c90_ (min)	CRI (min^−1^)
OPTB0	8.80	0.38	12.78	12.40	1.17	3.47	27.65
OPTB10	9.30	0.25	14.51	14.26	0.57	3.21	30.58
OPTB30	14.10	0.48	20.88	20.40	1.07	3.41	28.26
OPTB50	24.50	1.06	28.71	27.65	1.04	3.45	27.95
OPTB100	33.10	1.18	38.76	37.58	0.58	4.19	23.29

## Data Availability

The original contributions presented in this study are included in the article. Further inquiries can be directed to the corresponding author.
